# Effect of orthodontic forces on root length of immature mandibular second premolars: a split-mouth randomized clinical trial

**DOI:** 10.1590/2177-6709.26.5.e2119355.oar

**Published:** 2021-10-25

**Authors:** Kazem DALAIE, Mohammadreza BADIEE, Mohammad BEHNAZ, Shahab KAVOUSINEJAD

**Affiliations:** 1 Department of Orthodontics, School of Dentistry, Shahid Beheshti University of Medical Sciences, Tehran, Iran.; 2 Dentofacial Deformities Research Center, Research Institute of Dental Sciences, School of Dentistry, Shahid Beheshti University of Medical Sciences, Tehran, Iran.; 3 Postgraduate Student of orthodontics, Department of Orthodontics, Tehran University of Medical Sciences, Tehran, Iran.

**Keywords:** Immature teeth, Orthodontic force, Root formation, Root resorption

## Abstract

**Objective:**

To assess the effect of orthodontic forces on changes in root length of immature mandibular second premolars.

**Methods:**

Sixty-four mandibular second premolars (MSP) with immature apices (left and right sides) of 32 patients aged between 10 and 13 years were evaluated. Orthodontic treatment was started after obtaining periapical radiographs (T_1_) from the MSPs of each patient. Brackets were bonded, except the ones of MSPs (left or right by random as control MSP, and the other side as test MSP). After 9-12 months, a second periapical radiograph (T_2_) was obtained from the MSPs of each patient. Then, brackets were bonded to the control MSPs, which were not bonded before. After 18 ± 3 months, a third periapical radiograph (T_3_) was obtained. Changes in root length were evaluated by using a new formula. The test and control MSPs at T_1_, T_2_ and T_3_ were compared using repeated measures ANOVA and parametric tests. *P*-value smaller than 0.05 was statistically significant.

**Results:**

There was no significant difference between the test and control groups in the mean root length of MSP at T_1_ (*p*= 0.48) and T_3_ (*p*= 0.078). The root length at T_2_ (*p*= 0.001) was significantly different between test and control MSPs, and the test group showed longer root length than the control group.

**Conclusions:**

Orthodontic force applied for leveling and alignment of immature MSPs may not have destructive effects on the roots, and may accelerates root formation in short-term. Normal root length was achieved at the end of root development.

## RESUMO

**Objetivo:**

Avaliar o efeito das forças ortodônticas nas mudanças do comprimento radicular de segundos pré-molares inferiores com raízes incompletas.

**Métodos:**

Foram avaliados 64 segundos pré-molares inferiores (SPI) com raízes incompletas (lados direito e esquerdo da mandíbula) de 32 pacientes com idades entre 10 e 13 anos. O tratamento ortodôntico teve início após a obtenção de radiografias periapicais (T_1_) dos SPIs de cada paciente. Foram colados braquetes em todos os dentes, com exceção dos SPIs (do lado esquerdo ou direito, de forma aleatória, como grupo controle; e o SPI contralateral como grupo teste). Após 9 a 12 meses, uma segunda radiografia periapical (T_2_) foi obtida dos SPIs de cada paciente. Então, braquetes foram colados nos SPIs do grupo controle, que não haviam sido colados anteriormente. Após 18 ± 3 meses, uma terceira radiografia periapical (T_3_) foi obtida. As mudanças no comprimento radicular foram avaliadas por meio de uma nova fórmula. Os SPIs teste e controle foram comparados em T_1_, T_2_ e T_3_ usando ANOVA para medidas repetidas e testes paramétricos. Valores de *p*< 0,05 foram considerados estatisticamente significativos.

**Resultados:**

Não foi encontrada diferença significativa no comprimento radicular médio dos SPIs entre os grupos teste e controle em T_1_ (*p*= 0,48) e T_3_(*p*= 0,078). O comprimento radicular dos SPIs em T_2_ (*p*= 0,001) foi significativamente diferente entre os lados teste e controle, sendo que o grupo teste apresentou maior comprimento radicular do que o grupo controle.

**Conclusões:**

As forças ortodônticas aplicadas para o alinhamento e nivelamento de SPIs com raízes incompletas podem não ter efeitos danosos nas raízes, podendo, inclusive, acelerar a formação radicular em curto prazo. O comprimento radicular normal foi alcançado ao fim da formação radicular.

## INTRODUCTION

Advanced apical root resorption is a possible consequence of orthodontic treatment, which is also related to some biological factors.^1^ Shortening of root length as the result of external apical resorption is irreversible and unpredictable.^2^ The mean magnitude of root resorption varies from 0.5 mm to 3 mm, as reported in the literature.^3^ ^,^ ^4^ In the majority of orthodontic patients, the magnitude of root resorption is clinically irrelevant. In a limited number of patients, however, the teeth are severely affected. Resorption of more than half of the root length affects the function and compromises the survival of the teeth. A number of factors may play a role in this respect, including genetic susceptibility,^5^ ^,^ ^6^ thumb sucking,^7^ tooth root shape, history of trauma to the teeth prior to treatment onset, endodontic treatment, type of orthodontic appliance, the magnitude of load and tooth movement, duration of treatment and developmental stage of the root.^2^ In general, it has been well accepted that the late mixed dentition period and early permanent dentition period (at the time of eruption of canines and premolars) are the most suitable times for initiation of orthodontic treatment.^8^ At this time, the roots of the majority of permanent teeth have not yet fully developed.

The effect of orthodontic force on immature teeth with incomplete apex closure has not been adequately studied. Some authors^8^ ^,^ ^9^ believe that movement of teeth with open apex may serve as a risk factor for root resorption, short root length or early apex closure. Malformation of the Hertwig’s epithelial root sheath alters the trend of root apex calcification, and the root may not reach its normal length.^8^ ^,^ ^9^ Consolaro et al.^10^ reported that movement of open-apex teeth would decrease root length due to early apex closure, and not root resorption. On the other hand, some authors have claimed that teeth with undeveloped roots are more resistant to root resorption caused by orthodontic forces^7^ ^,^ ^11^ ^,^ ^12^ and tooth movement has no adverse effect on open-apex teeth.^13^ It was reported that orthodontic treatment did not affect dentin formation in young permanent teeth, or even activated odontoblasts and accelerated dentin mineralization.^14^ Mavragani et al.^4^ evaluated pre- and postoperative radiographs and demonstrated continuation of root development in lateral incisors with undeveloped roots during orthodontic treatment course, with no root shortening.

Periapical (PA) and panoramic radiographic images (OPG) can be used to assess root length during orthodontic treatment. Panoramic radiography has some limitations. For instance, the quality of a pantomograph (OPG) depends on the patient’s position and the distance between the anatomical structures and the focal trough.^15^ The magnification rate in different parts of the head and face is variable in OPG, ranging from 20 to 35%.^16^ However, the magnification rate in PA radiography is usually <5%.^17^ OPG is not suitable for assessment of root shape and other anomalies such as root dilaceration, since it can overestimate root resorption by 20%.^15^

Considering the high demand for orthodontic treatment in adolescents, the need for early initiation of orthodontic treatment and the existing controversies on the effect of orthodontic treatment on root development of open-apex teeth, the present study aimed to assess the effect of orthodontic treatment on root length of immature mandibular second premolars. Other studies evaluated the effect of orthodontic movement in immature incisors. In this study, posterior immature teeth (second premolars) were evaluated, and PA was the radiographic method applied.

## MATERIAL AND METHODS

### DESIGN

This randomized split-mouth clinical trial evaluated mandibular second premolars (MSPs). The mandibular second premolar of one side served as the test (test MSP), and the contralateral mandibular second premolar served as the control tooth (control MSP).

### PARTICIPANTS, ELIGIBILITY CRITERIA, AND SETTING

**Figure 1:**
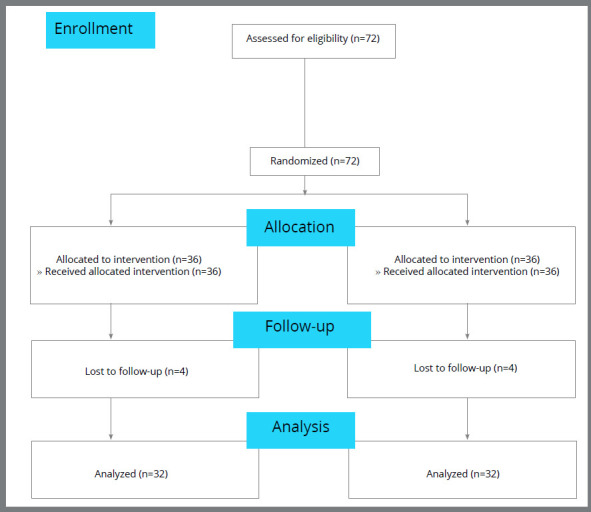
CONSORT flow diagram of the study.

Sixty-four mandibular second premolars of 32 orthodontic patients who required orthodontic treatment for mandibular anterior crowding were evaluated. The teeth had open apices, according to the primary panoramic radiograph. The patients were aged between 10 and 13 years, and were recruited from a dental clinic and two private offices in Tehran/Iran. All patients were treated by the same orthodontist. The inclusion criteria were: presence of MSP with immature roots (late F or early G stages, according to Demirjian^18^) in both the right and left sides of the mandible, the need for fixed orthodontic treatment, mild crowding (1-3 mm on each side) in the posterior mandible, Class I malocclusion, no caries or restorations in premolars, and no evidence of developmental dental anomalies before treatment. The exclusion criteria were root dilaceration, developmental dental anomalies and severe posterior crowding of the mandible (>4 mm). All patients were matched in terms of dental developmental stage (late F or early G, according to Demirjian^18^). The dental developmental stage of the MSPs on both sides of the mandible was the same. Figure 1 shows the CONSORT flow diagram of the study.

### INTERVENTION

At first, PA radiographs were obtained of the MSPs using the parallel technique (T_1_). Brackets (0.022-in slot MBT bracket system; American Orthodontics) were bonded to all teeth, except control MSP, which was randomly chosen to serve as the control group by using a table of random numbers. Leveling and alignment began with 0.014-in NiTi archwires (American Orthodontics). The sequence of wires was the same for all patients, and 0.018-in stainless steel wire was used after six months. At 9 months after the initiation of treatment, a second PA radiograph (T_2_) was obtained from the MSPs. Next, the brackets were bonded to the control MSP. From this stage until the end of treatment, both MSPs (test and control) used brackets and were subjected to force application. At the end of the treatment course (after 18 ± 3 months), a third PA radiograph was obtained (T_3_) from the MSPs. At this time, the MSPs had closed apices and their development had been completed. The sequence of archwires (T_1_ to T_3_) was: 0.014-in NiTi, 0.016-in NiTi, 0.016-in stainless steel, 0.018-in stainless steel, 0.016 x 0.022-in NiTi, 0.017 x 0.025-in NiTi and 0.018 x 0.025-in stainless steel for using finishing elastics. After bonding the control MSP, the previous sequence of NiTi archwires was replaced, which overlaid to the 0.018-in stainless steel archwire. The teeth did not have any root dilaceration or periapical lesion during or at the end of treatment. 

### PRIMARY AND SECONDARY OUTCOMES

**Figure 2:**
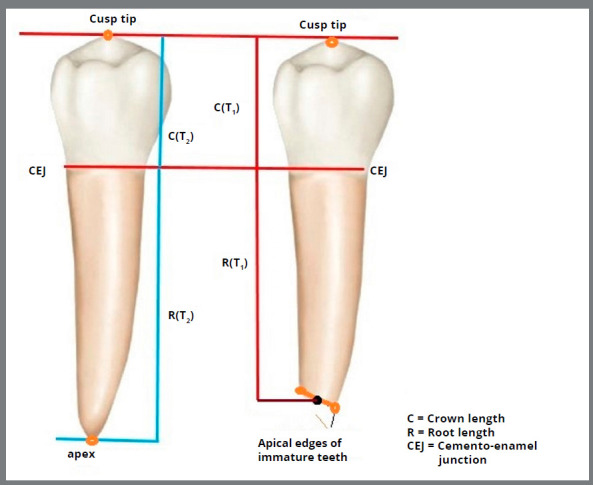
Schematic view of the measurements made for root length of mandibular second premolars on PA radiographs: (right) open apex, (left) closed apex.

The root length of MSPs was considered as the primary outcome, which was measured at three time points: T_1_ (baseline), T_2_ (after 9 to 12 months) and T_3_ (at the end of treatment, after 18 ± 3 months). Root length was measured on PA radiographs and compared between different time points. In other words, root length was compared between T_1_ and T_2_, and also T_1_ and T_3_, at the test and control sides. All PA radiographs were scanned digitally as an image and saved with the same resolution. Measurements were made using Photoshop software (Adobe^®^, USA). On each scanned image, four points - namely, the cusp tip, mesial and distal cementoenamel junction (CEJ), and root tip - were marked. Since the roots had not been fully developed, the root tip was considered as the point at the center of the line connecting mesial and distal edges of the root. The root length was measured from the root tip (for open apex teeth, the midpoint of the line connecting the mesial and distal edges of the apex was used) to the midpoint of the line connecting the mesial and distal CEJ (Fig 2).

**Figure 3:**
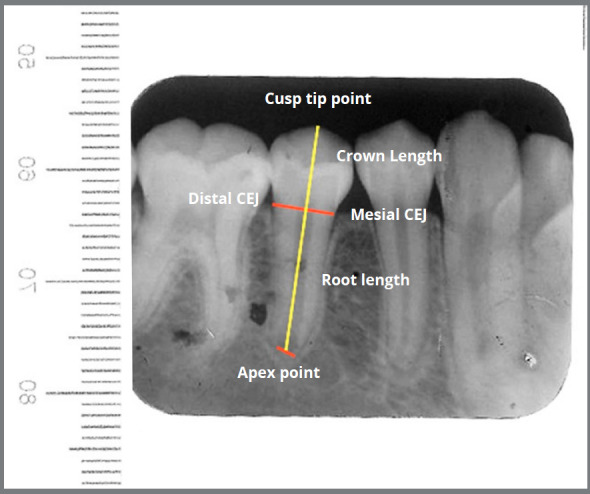
Measurement of crown and root length on digital scanned periapical radiographs with a transparent ruler to calibrate the image (production of pixel to millimeter conversion factor, PMCF).

**Figure 4:**
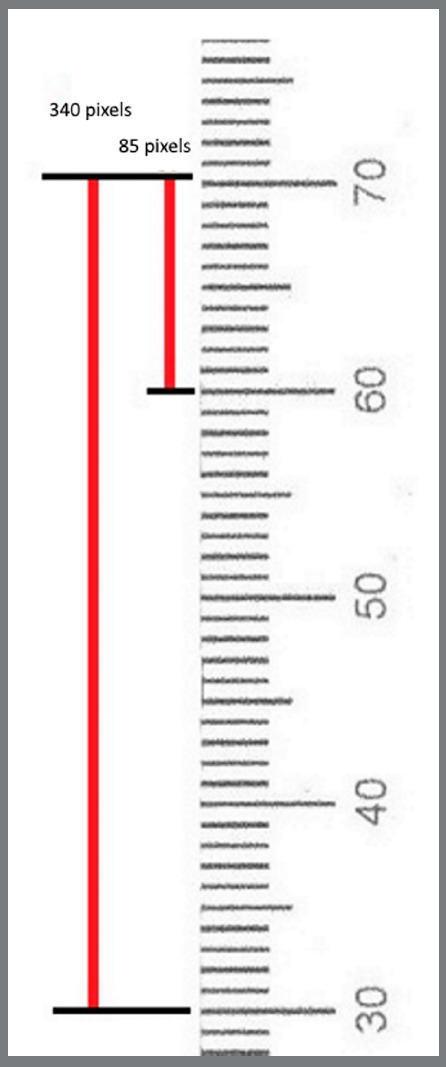
A transparent ruler scanned with PA digitally, showing how to calibrate the image (production of pixel to millimeter conversion factor, PMCF).

**Figure 5:**
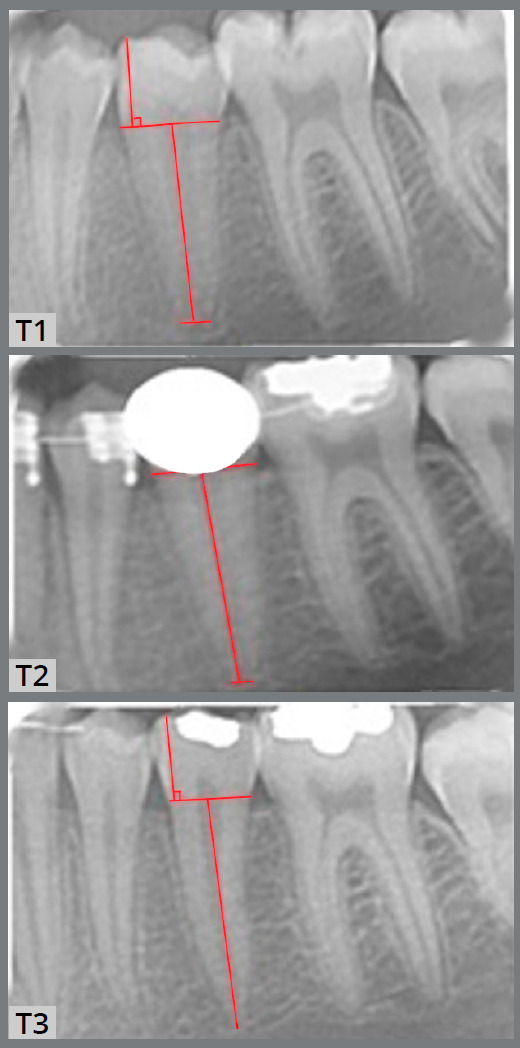
T1) The cusp tip, mesial and distal CEJ, and root tip were identified as reference points. The reference point of the root tip is the center of the line connecting the mesial and distal edges of the immature root. The length of the crown is the perpendicular distance of the crown tip to the mesial and distal CEJ connecting line. T2) Reference points and distances were identified. The mesial and distal CEJ are exactly similar to the T1 stage. Due to the development of the root, the root length is higher than T1. The second premolar crown was masked for blinding the study, but after the correction factor was calculated for T1 and T2, while CEJs are visible for measuring root length. T3) The reference points and distances were identified, the mesial and distal CEJ is exactly similar to the T1 and T2 stages. The root is mature in this stage and the apex is closed.

To reduce the digital image measurement error, two adjustment factors were applied. The pixel to millimeter conversion factor (PMCF) was applied for the T_1_, T_2_ and T_3_, and the correction factor (CF) was applied for T_2_ and T_3_ to minimize the measurement errors. To convert pixels to millimeter, all PA films were scanned with a transparent ruler (Fig 3). PMCF was calculated by dividing the digitally measured length on the ruler (e.g., 1 mm) by the number of pixels on the same length (e.g., measuring 1 mm on the ruler in the scanned film, in pixels) (Fig 4 and 5). For cases with difficulties in marking the cusp tip, another reference point on the crown was marked in T_2_ and T_3_ (for example, top of the restoration or somewhere on the crown). This cusp point should be constant in all the three images. The length of crown (from the same reference point in T_1_, T_2_ and T_3_) was used for calculating the correction factor (CF).

The following formula was used to calculate PMCF in millimeters per pixel (mm/pixel):

$$\;PMCF\;=\frac{\;Measured\;length\;}{\;Number\;of\;pixels\;in\;the\;same\;measured\;length\;}$$

The CF eliminates the possible difference between the T_1_ and T_2_, and also T_1_ and T_3_ radiographs. Since the crown length was constant, by dividing the crown length at T_1_ by the crown length at T_2_, the CF was obtained and multiplied by the root length values obtained at T_2_ and T_3_. The following formula was used to calculate CF:

$$CF2(\;for\;the\;\sec ond\;PA)=\frac{\;Crown\;length\;at\;T1}{\;Crown\;length\;at\;T2}$$

$$\;CF3\;(for\;the\;third\;PA)\;=\frac{\;Crown\;length\;at\;T1}{\;Crown\;length\;at\;T3}$$

To calculate CF, the same point at the tip of the crown was considered as the reference cusp tip on all three scanned PA radiographs. From this point, a line was drawn perpendicular to the line connecting the mesial and distal CEJ. The same process was repeated for all three PA radiographs of each patient. Next, the vertical length between the cusp tip and the line connecting the mesial and distal CEJ was measured in millimeters, and used in the CF calculation formula. Eventually, the CF for the comparison of T_2_ and T_1_ (CF2) and also T_3_ and T_1_ (CF3) was calculated for the right and left second premolars. The root length values at T_1_, T_2_ and T_3_ were placed in root length formula (Formulas 1 and 2) in millimeters. The exact value of change in root length was calculated by the formulas for each tooth (left and right mandibular second premolars). All measurements were made by a single operator. 

Comparison of T_2_ with T_1_ (Formula 1):

$$\;Change\;in\;root\;length\;(mm)=[PMCF\times CF2\times T_{2}rootlength(pixels)]-[PMCF\times T_{1}rootlength(pixels)]$$

Comparison of T_3_ with T_1_ (Formula 2):

$$\;Change\;in\;root\;length\;(mm)=[PMCF\times CF3\times T_{3}rootlength(pixels)]-[PMCF\times T_{1}rootlength(pixels)]$$

Negative values indicate root resorption, while positive values indicate root formation. After calculation of the root length in millimeters at T_1_, T_2_ and T_3_ in the right and left sides, the changes in root length were separately calculated for the test and control groups. The difference in second premolars root length was calculated for each group at T_2_ compared to T_1_, and at T_3_ compared to T_1_.

### SAMPLE SIZE CALCULATION

Significant difference in root length between the test and control groups was set at 0.4 mm (due to limitations in accurate measurement of length and risk of measurements errors on 2-D PA radiographs). All scanned radiographs were evaluated on a computer, with 382 x 304 pixels resolution. If the actual length of a standard PA radiograph (31 x 41 mm) is divided by the number of pixels in the same length on the scanned radiograph, the size of each pixel would be 0.107 mm. Thus, if the observer had 4 pixels of error (which is about 0.4 mm), this error in measurement could cause errors in calculations and the results.

### RANDOMIZATION (RANDOM NUMBER GENERATION, ALLOCATION CONCEALMENT, IMPLEMENTATION)

In this split-mouth clinical trial, the right and left MSPs were randomized into the test and control groups using a table of random numbers (simple randomization). Accordingly, at the onset of study, in 14 patients left MSP and in 18 patients right MSP were subjected to orthodontic force application (test group). 

### BLINDING

Patients were aware of the allocation of their teeth to the test or control group, but this knowledge had no significant effect on the intervention and was not a confounder. After obtaining the CF, the crown of all second premolars at T_2_ (in both the test and control groups) was masked, for blind measurement of root length (Fig 5). Thus, for measurement of root length at T_2_, it was not clear for the observer whether the second premolar belonged to the test (with bracket) or the control group (no bracket), and only the right and left side were noticeable. 

However, the mesial and distal CEJs were visible for root length measurement on all radiographs. Thus, this study had a single-blind design. 

### STATISTICAL ANALYSIS

Data were analyzed using SPSS v. 25 (SPSS Inc., IL, USA). Normal distribution of data was evaluated using the Kolmogorov-Smirnov and Shapiro-Wilk tests. Both tests confirmed normal distribution of data in both groups. Thus, the test and control groups were compared using repeated measures ANOVA and parametric tests. Multivariate ANOVA was applied to assess the effect of orthodontic force on changes in root length at the three time points. Using one sample *t*-test, the age of apex closure of second premolars in this study was compared with the average value.^19^ The reliability of measurements was evaluated by calculating the intra-examiner reliability, using the Cronbach’s alpha. For this purpose, 20 patients were randomly selected and measurements were repeated after 14 days, by the same examiner. The obtained values were compared with the baseline values. Statistical significance in this study was determined at *p* < 0.05.

### ETHICAL CONSIDERATIONS

The study was approved by the ethics committee of Shahid Behehsti University (IR.SBMU.RIOS.REC.1396.457), and registered at the Iranian Registry of Clinical Trials (IRCT20170928036471N1). All patients signed written informed consent forms prior to enrollment, and were briefed about the study.

## RESULTS

Of 32 patients evaluated in this study (Fig 1), 11 (34.4%) were males and 21 (65%), females. The mean age was 11.99 ± 0.75 years (ranging from 10.2 to 13.0 years). Table 1 shows the mean root length of second premolars in the two groups at T_1_, T_2_ and T_3_. Repeated measures ANOVA evaluated the effect of orthodontic force application (treatment), time (T_1_ to T_3_) and the interaction of both on root length, by taking into account the treatment and time as repeated factors. The interaction effect of time and treatment on root length was found to be statistically significant (*p*< 0.0001). Pairwise comparison of root length at the two sides at each time point showed a significant difference between the test and control groups at T_2_, after Bonferroni adjustment (*p*< 0.0001). 

Evaluation of the mean root length separately in each group between the two time points revealed a significant increase in root length over time. In other words, application of orthodontic force at T_2_ caused a significant increase in the mean root length, compared to the control group. Time had a significant effect on root length in both groups. With respect to treatment alone, the two groups had a significant difference at T_2_. Also, a significant correlation was noted between root length at T_1_, T_2_ and T_3_ time points. Root length increased from T_1_ to T_2_ and from T_2_ to T_3_ in both groups, irrespective of treatment. Regarding the interaction effect of time and treatment, the two groups were not significantly different at T_1_ (mean difference of 0.032 ± 0.044, *p*= 0.48) but a significant difference was noted between them at T_2_, when the root length in the test group (13.64 mm) was significantly higher than that of the control group (13.2 mm) (mean difference of 0.46 ± 0.042, *p*= 0.001). At T_3_, the two groups were not significantly different in the mean root length (mean difference of 0.063 ± 0.035, *p*= 0.078) (Table 1). 

**Table 1:** Mean root length of second premolars in millimeter (mm) in the two groups at T_1_, T_2_ and T_3_ (mandibular second premolar (MSP) at time 1, 2 and 3)

Time	Group	Minimum (mm)	Maximum (mm)	Mean (mm)	SD	Difference between test and control groups* (Mean ± SD in mm)	*p* **-value**
T_1_	Test MSP	11.03	13.90	12.38	0.66	0.032±0.044	0.48
	Control MSP	10.40	13.73	12.35	0.71		
T_2_	Test MSP	15.50	14.85	13.64	0.51	0.46±0.042	0.001
	Control MSP	12.00	14.40	13.17	0.54		
T_3_	Test MSP	13.06	15.90	14.42	0.63	0.063±0.035	0.078
	Control MSP	12.83	15.88	14.36	0.67		

Level of significance: p ≤ 0.05; *Pairwise comparison after Bonferroni adjustment. SD = standard deviation.

Multivariate ANOVA was applied to assess the effect of side of load application on root length, during application of orthodontic force, at the three time points. The results showed no significant association between side of load application and changes in root length (*p*= 0.94). 

One-sample *t*-test was used to compare the mean root length in the two groups at T_3_ and the normal root length of MSP, according to Wheeler’s Dental Anatomy.^20^ Table 2 showed no significant difference between the values of the test and control groups with the normal values (*p*= 0.535 for the test group, and *p*= 0.267 for the control group). To assess the intra-examiner reliability, the Cronbach’s alpha was calculated to be 0.99, which was considered excellent. One-sample *t*-test was applied to assess the difference between MSP apex closure age and the normal value reported in the Standards of Human Tooth,^19^ which revealed a significant difference in males (*p*= 0.007) but not in females (*p*= 0.267) (Table 3). However, no significant correlation was noted between sex and the age of MSP apex closure (*p*= 0.629). The measurement error between the first and the second (14 days later) measurements of 20 randomly patients was 0.33 ± 0.14 mm.

**Table 2:** Comparison of test and control groups in this trial with the normal root length of mandibular second premolar (MSP), according to Wheeler’s Dental Anatomy^20^.

	Root length (Mean ± SD in mm)	Difference with the normal root length of MSP according to Wheeler’s Dental Anatomy (mm)*	*p* **-value**
Normal root length of MSP according to Wheeler's Dental Anatomy	14.5	-	-
The test group in this trial	14.42 ± 0.63	- 0.08	0.535
The Control group in this trial	14.36 ± 0.67	- 0.14	0.267

Level of significance: p ≤ 0.05; *One-sample t-test. SD = standard deviation.

**Table 3:** Difference in mandibular second premolar (MSP) apex closure age with the normal value reported in the Standards of Human Tooth (Smith^19^, 1991).

Sex	MSP apex closure mean age in this trial (Mean ± SD in years)	Standard mean age of MSP apex closure according to Standards of Human Tooth (in years)	*p* **-value**
Male	13.44 ± 0.31	14.3±0.11	0.007
Female	13.5 ± 0.15	13.7±0.13	0.267

Level of significance: *p* ≤ 0.05; *One-sample *t*-test. SD = standard deviation.

### HARMS

Premolar teeth were not harmed in this study. Repeated PA radiographs may be the only harm in this trial, but this is less than OPG X-ray exposure.^21^ OPG has some disadvantages, such as higher magnification rate than PA and measurement errors in the digital image of OPG.^15^ ^,^ ^16^

## DISCUSSION

This study assessed the effect of orthodontic force application on root length of immature teeth (mandibular second premolars, MSP) of 10 to 13-year-old patients. This study used a split-mouth design because individual differences could have affected the results. Thus, in each patient, the MSP of one side served as the test, and the MSP of the other side served as the control group. The results showed that in short-term, application of orthodontic force increased the root length of immature MSPs and the teeth reached their normal root length at the end of treatment. This finding is in contrast to those of Consolaro et al.^10^, Oppenheim^8^ and Phillips,^9^ who showed that application of orthodontic force prevented the immature teeth to reach their normal root length, probably due to early apex closure that occurs following enhanced calcification because of deformation of Hertwig’s epithelial root sheath. The difference between those and the present results may be attributed to more precise methodology and measurement method in the present clinical trial, compared to the other studies. 

More recent studies, in line with the present findings, indicated that orthodontic movement of immature teeth had no adverse effect on root development.^2^ ^,^ ^22^ Sameshima and Sinclair^13^ observed that immature teeth were more resistant to root resorption when orthodontic force was applied. Mavragani et al.^4^ concluded that final root length of teeth immature at treatment onset was significantly larger than that of developed roots. Accordingly, they suggested initiation of orthodontic treatment at a young age.^4^ Xu and Baumrind^23^ indicated low risk of root resorption in immature teeth. More recent studies confirmed no adverse effect of orthodontic force on immature teeth. For instance, Hendrix et al.^2^ showed that immature teeth were more resistant to root resorption during orthodontic treatment, and suggested completion of orthodontic treatment before root completion. Sameshima and Sinclair^13^ reported the same results, and Silva Filho et al.^24^ indicated normal development of a permanent central incisor with immature root, following orthodontic movement. However, they only evaluated roots length qualitatively. 

Rudolph^25^ discussed that early orthodontic treatment has less destructive effects on teeth. Andreasen et al.^26^ in an animal study demonstrated that trauma to the Hertwig’s epithelial root sheath had no adverse effect on root development. Thus, it seems that trauma due to application of orthodontic force does not adversely affect the performance of Hertwig’s epithelial root sheath. Kong et al.^14^ showed that application of orthodontic force resulted in greater activity of odontoblasts and formation of a thicker predentin layer. Reitan^27^ indicated that apical root resorption did not prevent root development, due to the presence of relatively thick predentin, which was non-calcified and was therefore not influenced by clastic cells. The present results confirmed their histopathological findings, since in short-term (9 months after treatment onset), teeth under orthodontic force application had a higher root length than control teeth. In long-term (18 months after treatment onset), the root lengths of the test and control teeth were the same. There is a possibility that application of orthodontic force enhances the process of root formation, but this process has no significant effect on normal root development. From T_1_ to T_2_, one of the MSPs (left or right, by random) of each patient was not under force. From T_2_ to T_3_, both of them were under force, and the apex was still immature. At T_3_, the apex was closed. From T_2_ to T_3_, orthodontic force influenced on both groups. Note that during T_1_ to T_2_, orthodontic force may have accelerated the root formation in the experimental group (according to the results of this study). The experimental MSPs were under orthodontic force sooner than the control MSPs, for evaluating the difference in amount of root formation in a period of time.

Stenvik and Mjo^28^ stated that dislocation of the Hertwig’s epithelial root sheath can cause dilaceration during orthodontic movement of immature teeth. In the present clinical trial, none of the teeth that were subjected to leveling and alignment had dilaceration at the end of treatment, despite the fact that they were at the late F or early G stage, according to Demirjian.^18^ This finding may be due to developmental stage of the teeth, and small magnitude of movement because of mild crowding (1 to 3 mm) in the posterior region. Thus, developmental stage of teeth, magnitude of tooth movement and the magnitude of orthodontic force play a role in this respect. Orthodontic force applied to teeth in earlier developmental stages increases the risk of dilaceration. Also, increasing the magnitude of orthodontic tooth movement would increase the deformation of Hertwig’s epithelial root sheath and subsequent root apex dilaceration. Moreover, teeth with immature apices would experience less pulpal changes (such as changes in blood supply). Since the dental pulp of immature teeth is less affected by changes in blood supply during application of orthodontic forces, they have higher tolerance threshold than teeth with closed apices.^29^ It appears that the tissue surrounding an immature root can protect the mineralized tissue against resorption during orthodontic treatment, and allow continuation of root formation and maturation. 

Considering all the aforementioned, as well as the findings of the current study, it may be stated that orthodontic force application does not cause shortening of the roots; instead, it enhances root formation, at least in short term. 

The limitation of this study is that the accuracy of measurements on 2-D digital radiographs is lower than that of 3-D radiographs. Another limitation was the difficulty in marking a point on the crown of some MSPs, due to restorations, rotations and etc. Further studies are required on the effect of other tooth movements, such as bodily movement and rotation, on root formation. Evaluation of blood flow in the periodontium around the apex of immature teeth during orthodontic treatment can be an interesting topic of research for future studies as well. 

## CONCLUSION

Orthodontic force applied for leveling and alignment of immature teeth may not have destructive effects on the roots, and even accelerates root formation in short-term. Normal root length is achieved at the end of root development.
